# SCARF1 promotes M2 polarization of Kupffer cells via calcium‐dependent PI3K‐AKT‐STAT3 signalling to improve liver transplantation

**DOI:** 10.1111/cpr.13022

**Published:** 2021-03-09

**Authors:** Xue‐song Xu, Zhi‐hao Feng, Ding Cao, Hao Wu, Meng‐hao Wang, Jin‐Zheng Li, Jian‐Ping Gong

**Affiliations:** ^1^ Department of Hepatobiliary Surgery The Second Affiliated Hospital of Chongqing Medical University Chongqing China

**Keywords:** Kupffer cells, liver transplantation, M2 polarization, phagocytosis, SCARF1

## Abstract

**Objectives:**

This study aimed to investigate the protective effect of SCARF1 on acute rejection (AR), phagocytic clearance of Kupffer cells (KCs), M2 polarization and the exact mechanism underlying these processes.

**Methods:**

AAV was transfected into the portal vein of rats, and AR and immune tolerance (IT) models of liver transplantation were established. Liver tissue and blood samples were collected. The level of SCARF1 was detected via WB and immunohistochemical staining. Pathological changes in liver tissue were detected using HE staining. Apoptotic cells were detected using TUNEL staining. KC polarization was assessed via immunohistochemical staining. Primary KCs were isolated and co‐cultured with apoptotic T lymphocytes. Phagocytosis of apoptotic cells and polarization of KCs were both detected using immunofluorescence. Calcium concentration was determined using immunofluorescence and a fluorescence microplate reader. The levels of PI3K, p‐AKT and P‐STAT3 were assessed via WB and immunofluorescence.

**Results:**

Compared to the IT group, the level of SCARF1 was significantly decreased in the AR group. Overexpression of SCARF1 in KCs improved AR and liver function markers. Enhanced phagocytosis mediated by SCARF1 is beneficial for improving the apoptotic clearance of AR and promoting M2 polarization of KCs. SCARF1‐mediated enhancement of phagocytosis promotes increased calcium concentration in KCs, thus further activating the PI3K‐AKT‐STAT3 signalling pathway.

**Conclusions:**

SCARF1 promotes the M2 polarization of KCs by promoting phagocytosis through the calcium‐dependent PI3K‐AKT‐STAT3 signalling pathway.

## Graphical abstract

Upregulation of SCARF1 in KCs to enhance its phagocytosis of apoptotic cells increased intracellular calcium efflux, and then up‐regulated PI3K‐AKT‐STAT3 signal activity, and ultimately promoted the M2 polarization of KCs; at the same time, it decreased the secretion of pro‐inflammatory factors, increased the secretion of anti‐inflammatory factors, which was conducive to the formation of immune tolerance after liver transplantation.

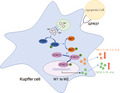

## 1 INTRODUCTION

Acute rejection (AR) and its related complications remain one of the most important factors affecting the long‐term survival of patients who have undergone liver transplantation. The elimination of apoptotic cells is believed to play a role in the formation of a microenvironment supportive of immune tolerance (IT).^1^, ^2^ During inflammation, the timely elimination of apoptotic cells can promote the secretion of anti‐inflammatory factors such as IL‐10 and TGF‐β and can induce the production of regulatory T lymphocytes. Recently, the function of SCARF1, a type of PtdSer receptor, has been revealed. It is speculated that SCARF1 plays an important role in the elimination of apoptotic cells and in the formation of a microenvironment supportive of IT.^3^

Kupffer cells (KCs) are a type of non‐parenchymal liver cell. Due to their high plasticity, KCs can play various roles in different microenvironments.^4^, ^5^, ^6^ KCs possessing the M1 phenotype can secrete pro‐inflammatory factors, present antigens and positively regulate immune rejection. KCs possessing an M2 phenotype primarily promote the secretion of anti‐inflammatory factors and function to negatively regulate immune rejection. Therefore, remodelling the phenotype of KCs towards the M2 phenotype benefits the formation of a microenvironment supportive of IT. According to a previous study,^7^ following the phagocytosis of apoptotic cells induced by SCARF1, antigen‐presenting cells (APCs) can produce large amounts of IL‐10 in a manner similar to that of M2 phenotype KCs. Based on this, we believe that SCARF1 may participate in the regulation KC transformation that may be related to the enhanced phagocytosis of APCs mediated by SCARF1. However, the exact mechanism underlying this process remains to be further studied.

It is believed that the activation of STAT3 plays an important role in the elimination of apoptotic cells and the formation of an IT microenvironment.^8^, ^9^, ^10^ STAT3 also participates in the regulation of macrophage functions, particularly in immune function.^11^ According to a previous study,^12^ the different regions of STAT3 possess different functions. In response to different post‐transcriptional modifications, the activation of STAT3 can be altered to adapt to the local microenvironment. Post‐transcriptional modifications of STAT include phosphorylation, acetylation and methylation. Among these modification types, phosphorylation of STAT3 is the most common. In response to changes in phosphorylation status, STAT3 participates in the regulation of different physiological functions.

Previous studies have found that the PI3K‐AKT signalling pathway plays an important role in the regulation of macrophage polarization, in particular by affecting the phosphorylation level of STAT3.^13^, ^14^ However, the PI3K‐AKT signalling pathway itself is affected by changes in cytoplasmic calcium concentrations.^15^, ^16^ Notably, the intracellular calcium concentration was significantly increased during phagocytosis by APCs to clear apoptotic cells.^17^ Based on the above study, we hypothesized that by recognizing apoptotic T lymphocytes, KCs can eliminate apoptotic T lymphocytes and transform into the M2 phenotype due to changes in STAT3 phosphorylation. Following this phenotype transformation, a large number of anti‐inflammatory factors can be secreted and the microenvironment of IT can be formed. During this process, the SCARF1‐mediated calcium‐dependent PI3K‐AKT‐STAT3 signalling pathway may play an important role.

In our study, we investigated the effect of upregulation of SCARF1 onKCs and on AR of the liver to further elucidate possible mechanisms underlying these processes and to aid in identifying new and efficient treatments to induce the formation of a microenvironment supportive of liver IT.

## 2 MATERIALS AND METHODS

### 2.1 Materials

AAV‐CD68‐MCE‐EGFP‐3Falg and AAV‐CD68‐MCE‐SCARF1‐EGFP‐3Falg were obtained from Genechem Company Ltd. (Shanghai). Dulbecco's modified Eagle's medium (DMEM) was obtained from Abcam Trading (Shanghai) Co. Ltd. (Shanghai, China). Enzyme‐linked immunosorbent assay (ELISA) kits for IL‐1β, TNF‐α and IL‐6 were obtained from Abcam Trading (Shanghai) Co. Ltd. (Shanghai, China). Primers for SCARF1 were provided by Sangon Biotech (Shanghai, China) Co. Ltd (Shanghai, China). The terminal deoxynucleotidyl transferase‐mediated dUTP‐biotin nick‐end labelling (TUNEL) kit was supplied by Roche Company (Shanghai, China). Antibodies against SCARF1, CD86 and iNOS were purchased from Proteintech Group. Co. Lid (Wuhan, China). Antibodies against cleaved‐caspase3, caspase3, Bax, Bid, CD206, CD163, PI3K, p‐STAT3 and STAT3 were purchased from Abcam Trading Company Ltd. (Shanghai, China). AKT, p‐AKT and GAPDH were purchased from Cell Signaling Technology Co. Ltd. (Shanghai, China). The relevant antibody information is provided in Table 1. Fluo‐4AM was purchased from Beyotime Biotechnology. Co. Lid (Shanghai, China). T lymphocytes were purchased from the Cell Bank of the Chinese Academy of Sciences (Shanghai, China). All other reagents used in this study were commercially available and were of analytical grade.

**TABLE 1:** The relevant antibody information

Name	Catalogue numbers	Company
SCARF1	13702‐1‐AP	Proteintech Group
c‐caspase3	ab49822	Abcam
caspase3	ab184787	Abcam
Bax	ab182733	Abcam
Bid	ab272880	Abcam
CD206	ab64693	Abcam
CD163	ab182422	Abcam
CD86	13396‐1‐AP	Proteintech Group
iNOS	18985‐1‐AP	Proteintech Group
PI3K	ab191606	Abcam
p‐AKT	#4685	CST
AKT	#4058	CST
STAT3	ab68153	Abcam
p‐STAT3	ab32143	Abcam
GAPDH	#5174	CST

### 2.2 Animals and protocols

All the male BN and Lewis rats (male, 8‐10 weeks old, each weighing 285 ± 42.6 g) were provided by the Experimental Animal Center of Chongqing Medical University (Chongqing, China). Humane care guided by the National Institutes of Health was provided to all animals. The protocols used in this research were evaluated and approved by the Animal Use and Ethic Committee of the 2nd Affiliated Hospital of Chongqing Medical University (2018‐2021). To more accurately compare the difference between AR and IT after liver transplantation, the IT model of liver transplantation was selected as the control. Next, BN and Lewis rats were randomly paired and divided into four groups as follows:

1. IT group (n = 30), in which rats underwent BN‐to‐BN orthotopic liver transplantation to establish the IT model. As there was no significant difference in pathological changes in liver tissue in the IT group with or without AAV transfection, we did not use IT + Ctrl or IT + OE groups in the follow‐up experiment (Figure S1).
2. AR group (n = 30), in which rats underwent BN for Lewis orthotopic liver transplantation to establish the AR model.
3. AR + Control‐AAV (AR + Ctrl) group (n = 30), in which rats underwent BN for Lewis orthotopic liver transplantation to establish the AR reaction (AR) model. Two weeks prior to the operation, donor rats were injected through the portal vein with AAV_9_‐CD68‐MCE‐EGFP‐3Falg (0.5 mL, 2.5 × 10^13^ PFU/mL).
4. AR + overexpression‐AAV (AR + OE) group (n = 30), in which rats underwent BN to Lewis orthotopic liver transplantation to establish the AR model. Two weeks prior the operation, donor rats were injected with AAV_9_‐CD68‐MCE‐ SCARF1‐EGFP‐3Falg (0.5 mL, 2.5 × 10^13^ PFU/mL) through the portal vein.

Two weeks after transfection, cell‐specific identification of AAV transfection was detected according to living fluorescence imaging. The overexpression effect was also detected using RT‐PCR and immunofluorescence staining. As shown in Figure S2A, the fluorescence intensity in liver tissue was significantly higher than that in other tissues (R). However, after blocking KCs in liver tissue by treatment with GdCl_3_, the fluorescence intensity in liver tissue decreased significantly (L). As shown in Figure S2B, the level of SCARF1 mRNA in KCs in the AR + OE group was significantly higher than that in the AR group or the AR + Ctrl group. As shown in Figure S2C, the number of SCARF1‐positive KCs (level of co‐localization of red fluorescence and green fluorescence) was significantly higher than that in the AR group or the AR + Ctrl group. This suggests that our transfection method can effectively overexpress SCARF1 in KCs.

### 2.3 Isolation, cultivation and function identification of KCs

According to the three‐step approach proposed by Li ^18^ that included digestion by collagenase IV, gradient centrifugation and selective adherence, KCs were isolated from normal liver samples. KCs were then cultured in DMEM supplemented with 10% FBS, 100 U/mL penicillin G and 100 U/mL streptomycin at 37°C in the presence of 5% CO_2_. After culturing for 24 hours, the phagocytosis of KCs was examined using the ink assay, and the surface molecules of KCs were detected via immunofluorescence staining (Figure S3). As shown in Figure S3A, at 2 hours after culture, KCs were round and adhered well to the culture dish. As shown in Figure S3B, at 24 hours after culture, the morphology of KCs was typical fusiform. As shown in Figure S3C, numerous ink particles were observed in the KCs. As shown in Figure S3D, the percentage of F4/80‐positive cells was greater than 90%. This indicates that our method can efficiently separate KCs from liver tissue while retaining normal phagocytosis.

### 2.4 Co‐culture of KCs and apoptotic T lymphocyte

T lymphocytes were cultured in DMEM supplemented with 10% FBS, 100 U/mL penicillin G and 100 U/mL streptomycin at 37°C in the presence of 5% CO_2_ for 24 hours. T lymphocytes were treated with 800 µM H_2_O_2_ at room temperature for 1 hour to obtain apoptotic T lymphocytes. Next, the level of T cell apoptosis was detected using flow cytometry (Figure S4). After 800 µM H_2_O_2_ treatment, the level of T cell apoptosis was greater than 95%, indicating that our induction method was effective. KCs were then co‐cultured with apoptotic T lymphocytes at a ratio of 10:1.

### 2.5 Histopathological examination

Liver samples from the different groups were collected after surgery. The liver tissues were fixed with 10% buffered formaldehyde for 24 hours. Next, all samples were embedded in paraffin, sectioned and stained with haematoxylin and eosin. The histopathological changes were observed using an inverted microscope, and the hepatic damage in each group was evaluated using the Banff score. According to the rejection activity index (RAI), scores ranging from 0 to 3 were regarded as uncertain for AR, scores ranging from 3 to 5 were regarded as mild AR, scores ranging from 5 to 7 were regarded as moderate AR, and scores ranging from 7 to 9 were regarded as severe AR.

### 2.6 Examination of liver function

Blood samples from each group were collected after the operation. The serum liver function markers, including alanine aminotransferase (AST) and aspartate transaminase (ALT), were detected using an automatic biochemical analyzer (Beckman CX7, Beckman Coulter, CA, USA) to evaluate liver function.

### 2.7 Real‐time reverse transcription‐polymerase chain reaction (qRT‐PCR)

#### 2.7.1 Total RNA extraction

Total RNA was extracted as follows:

1. Samples were immerged in TRIzol reagent (Takara, Japan) on ice for 10 minutes at room temperature. The suspensions were then centrifuged at 4°C and 7992 *g* for 5 minutes. Sediments were discarded.
2. The suspensions were immerged in CCl4 at room temperature for 15 minutes. The suspensions were then centrifuged at 4°C and 12 000 rpm for 5 minutes. Sediments were discarded.
3. The suspensions were immerged in isopropanol at room temperature for 20 minutes. Then, suspensions were centrifuged at 4°C and 12 000 rpm for 10 minutes. Sediments were retained.
4. The sediments were immerged in ethyl alcohol and then centrifuged at 4°C and 12 000 rpm for 5 minutes. Then, sediments were dissolved in DEPC.

#### 2.7.2 Reverse transcription

1. The reaction system 1 was prepared (5× gDNA Eraser Buffer 2.0 μL, gDNA Eraser 1.0 μL, total RNA 2 μL, RNase‐free dH2O to 10 μL) on ice. Reaction system 2 was prepared (RT Primer Mix 1.0 μL PrimeScript RT Enzyme Mix I 1.0 μL, RNase‐Free dH2O 4.0 μL, reaction system 1 10.0 μL, 5× PrimeScript Buffer2 4.0 μL) on ice.
2. The reverse transcription was performed at 37°C for 15 minutes and 85°C for 5 seconds. Then, product was then cooled and saved at 4°C

#### 2.7.3 qRT‐PCR

1. Reaction system consisted of mRNA 2 mL, forward primer 1 μL, reverse primer 1 μL, SYBR^®^Premix Ex Taq II 12 mL and RNase‐free dH2O to a volume of 25 µL.
2. qRT‐PCR was performed as follows: initial denaturation at 95°C for 5 minutes, denaturation at 94°C for 60 seconds, annealing at 58°C for 69 seconds and extension at 72°C for 60 seconds for 40 cycles.

All samples were normalized according to GAPDH expression.

### 2.8 Western blot analysis

#### 2.8.1 Total protein extraction

The total protein was extracted as follows:

1. Radioimmunoprecipitation assay buffer containing phenyl‐methane‐sulfonyl fluoride (100 M) and sodium fluoride (100 M) was added to the suspension on ice for 30 minutes.
2. The suspension was centrifuged at 4°C and 12 000 rpm for 5 minutes. The loading buffer was added, and then, the suspension was heated at 95°C for 10 minutes.

Then, the total protein was stored at 4°C.

#### 2.8.2 Electrophoresis, membrane transfer and blocking

1. The protein samples were electrophoresed in 10% sodium dodecyl sulphate‐polyacrylamide gels at 80 V for 40 minutes and then at 100 V for 2 hours.
2. The polyvinylidene difluoride (PVDF) membrane was immersed in methyl alcohol for 5 minutes. The proteins were then transferred onto the PVDF membranes at 100 V for 2 hours.
3. The PVDF membranes were blocked with 5% bull serum albumin (BSA) for 1 hour.

#### 2.8.3 Incubation and detection

1. The PVDF membranes were incubated at 4°C overnight with primary antibodies targeting SCARF1 (1:500), c‐caspase3 (1:1000), caspase3 (1:1000), Bax (1:1000), Bid (1:1000), CD206 (1:1000), CD163 (1:1000), iNos (1:500), CD86 (1:500), PI3K (1:1000), p‐AKT (1:1000), AKT (1:1000), p‐STAT3 (1:1000), STAT3 (1:1000) and GAPDH (1:3000).
2. PVDF membranes were immersed in phosphate‐buffered saline solution with 0.1% Tween 20 for 5 minutes. Next, the PVDF membranes were incubated at room temperature with the appropriate secondary antibody (1:5000) for 1 hour.
3. The signals were detected via chemiluminescence using a gel imaging system. The relative expression of the proteins of interest was normalized to GAPDH expression.

### 2.9 Immunostaining for TUNEL

TUNEL assays were performed on the paraffin sections of the hepatic tissues using the TUNEL kit to assess the number of apoptotic T lymphocytes. The paraffin sections were dewaxed with xylene, gradient eluted using ethyl alcohol and successively immersed in working buffer and blocking buffer. Next, TdT and streptavidin‐HRP were successively added to each sample to complete the staining. The staining of each group of cell nuclei was observed using fluorescence microscopy. Green cell nuclei were identified as TUNEL‐positive cells.

### 2.10 Immunohistochemical staining

The paraffin sections were dewaxed using xylene and then gradient eluted with ethyl alcohol. After dewaxing and elution, the sections were digested with pancreatic enzymes at 37°C for 30 minutes. Next, the sections were boiled in citrate buffer solution for 5 minutes. The paraffin sections were blocked with goat serum at 37°C for 10 minutes, incubated with primary antibody against SCARF1 (1:100), CD163 (1:200) and iNOS (1:200) at 4°C overnight and then incubated with secondary antibody at 37°C for 30 minutes. Colour liquid (including DAB, H_2_O_2_ and PBS) was then added to the sections for staining. Next, haematoxylin was added to the sections at room temperature for 30 seconds. Finally, the paraffin sections were dewaxed using xylene and again gradient eluted with ethyl alcohol. The staining of each section was observed using inverse microscopy.

### 2.11 Immunofluorescence staining

Each group of KCs was fixed by immersing in 4% buffered formaldehyde at room temperature for 10 minutes and then permeated by 0.2% Triton X100 at room temperature for 30 minutes and blocked with 1% BSA at room temperature for 30 minutes. KCs were then washed with PBS at room temperature for 5 minutes. KCs were next incubated and protected from light at 4°C overnight with primary antibodies against CD163 (1:200), F4/80 (1:200) and STAT3 (1:200). Following this, KCs were washed and protected from light at room temperature for 5 minutes. KCs were then incubated and protected from light with a secondary antibody (1:1000) at room temperature for 1 hour. KCs were next washed and protected from light at room temperature for 5 minutes. Finally, KCs were incubated and protected from light with DAPI at room temperature for 10 minutes or Hoechst at 37°C for 30 minutes. After sealing using a fluorescent quenching agent, the staining of KCs was observed through the use of fluorescence microscopy.

### 2.12 Analysis using flow cytometry

The degree of apoptosis in T lymphocytes was detected via flow cytometry using the Annexin V‐FITC/PI apoptosis detection kit.

### 2.13 ELISA

ELISA was performed to detect the levels of TNF‐α, IL‐6 and IL‐10 in blood. Antigen serum was added to an ELISA plate (100 μL/well) overnight. After 24 hours, the ELISA plate was washed with PBS for 30 minutes and then air dried. Samples were added to an ELISA plate (100 μL/well) at 37°C for 1 hour. After 1 hour, the ELISA plate was washed with PBS. Next, antibody against IgG was added to the ELISA plate for 1 hour. After 1 hour, the ELISA plate was wished with PBS. Subsequently, working solution was added to the ELISA plate at 37°C for 30 minutes. Finally, a colour reagent was added to the ELISA plate. The index of each plate was detected using ELISA, and the levels of TNF‐α, IL‐6 and IL‐10 in serum were also detected using ELISA.

### 2.14 Statistical analysis

All results were analysed using SPSS 18.0 software (SPSS Inc, Chicago, USA). Normally distributed data are shown as mean ± SD. Differences between groups were detected using a t test. The Shapiro‐Wilk test was used to test normality. Data exhibiting a significance value > .05 were regarded as conforming to a normal distribution. Non‐normally distributed data are shown as the median, and differences were detected using the rank‐sum test. Differences with *P* values < .05 were regarded as statistically significant.

## 3 RESULTS

### 3.1 SCARF1 was decreased in KCs during AR after liver transplantation

**FIGURE 1:**
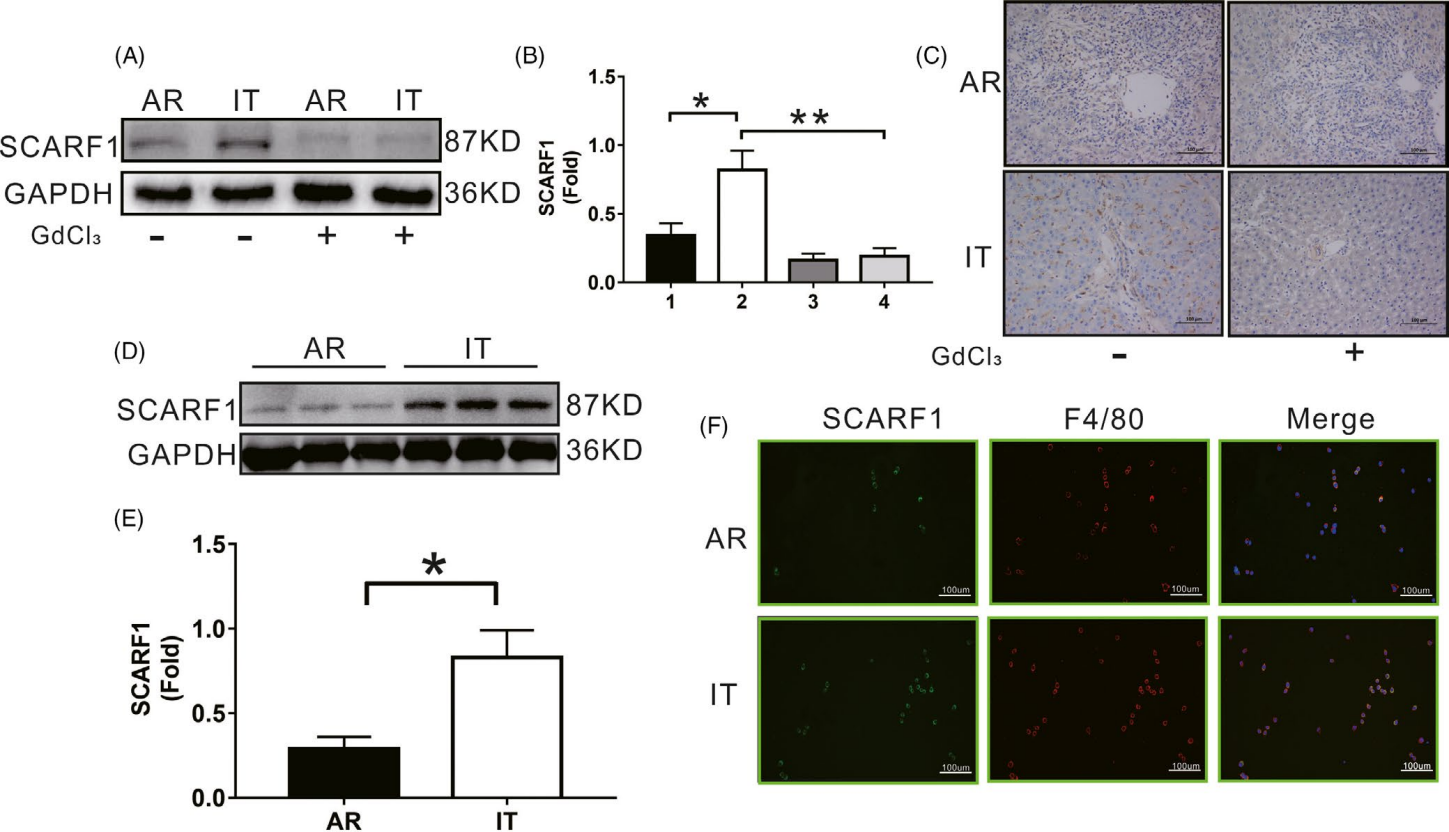
SCARF1 expression in the liver after transplantation. A, SCARF1 expression in the AR group and the IT group in the presence or absence of GdCl_3_ was detected via WB. B, Relative expression of SCARF1 in each group, 1: AR group, 2: IT group, 3: AR + GdCl_3_ and 4: IT + GdCl_3_. C, The SCARF1 expression in each group was detected via immunohistochemistry (200×). D, The SCARF1 expression of KCs was detected via WB in each group. E, Relative expression of SCARF1 in each group. F, The co‐localized expression of F4/80 and SCIMP in KCs was detected via immunofluorescence in each group (200×). *: *P* < .05, **: *P* < .01

To investigate whether SCARF1 is involved in the progression of liver transplantation AR, the expression of hepatic SCARF1 was detected in the AR and IT groups. As shown in Figure 1A,B, compared to that of the IT group, the expression of hepatic SCARF1 in the AR group was significantly decreased. Meanwhile, SCARF1‐positive areas in the AR group were significantly lower than those in the IT group (Figure 1C). It should be noted that both WB and immunohistochemical results demonstrated that the expression of SCARF1 in the liver was significantly decreased after KCs were depleted using GdCl_3_, particularly in the IT group. These results preliminarily suggest that SCARF1 in KCs may be involved in the process of acute liver transplantation rejection. We further identified the cell origin of SCARF1 in the liver. KCs were isolated from liver tissues in the AR and IT groups. The expression of SCARF1 in KCs from the AR group was significantly lower than that in the IT group (Figure 1D,E). The KCs in the AR and IT groups were then labelled with F4/80 (red). We observed that the expression of SCARF1 (labelled by green) in the AR group was significantly lower than that in the IT group (Figure 1F). Our results revealed that SCARF1 is expressed primarily in KCs and may be important in the context of acute liver transplantation rejection.

### 3.2 SCARF1 overexpression in KCs alleviated AR after liver transplantation

**FIGURE 2:**
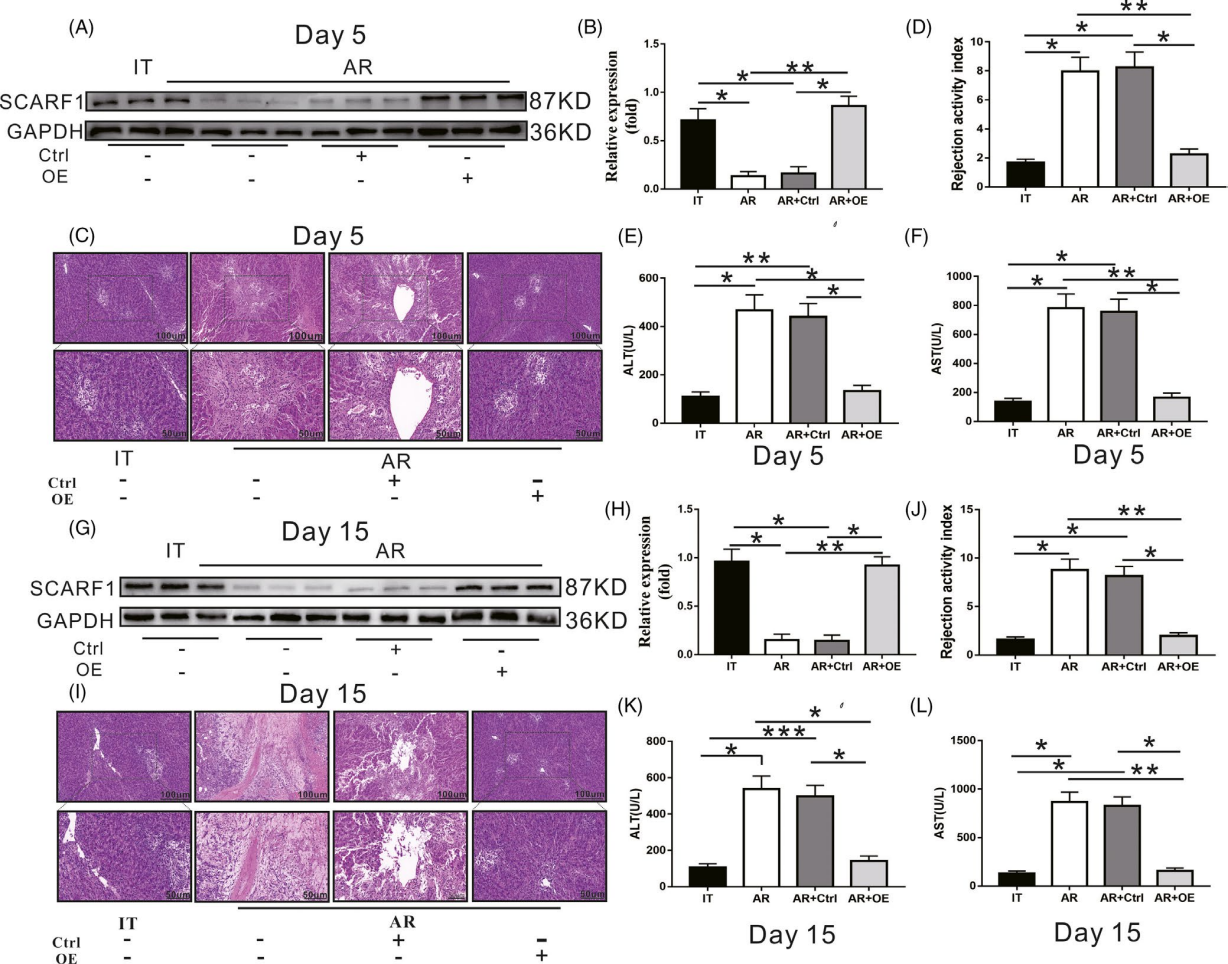
Effects of SCARF1 overexpression on acute rejection (AR) and liver function markers in the context of liver transplantation. (A‐F: 5 days after liver transplantation). A, SCARF1 expression in each group was detected via WB. B, Relative expression of SCARF1 in each group. C, AR of liver tissue in each group was detected via HE staining (up: 200×, down: 400×). D, AR index of each group. E and F, the levels of ALT and AST in each group were detected using an automatic biochemical analyzer. (G‐L: 15 days after liver transplantation). G, The SCARF1 expression in each group was detected via WB. H: Relative expression of SCARF1 in each group. I, AR of liver tissue in each group was detected via HE staining (up: 200×, down: 400×). J, AR index of each group. K and L, Levels of ALT and AST in each group were detected using an automatic biochemical analyzer. *: *P* < .05, ***: *P* < .01

As the expression of SCARF1 in KCs was significantly decreased in the AR model after liver transplantation, we used CD68 labelled adeno‐associated virus to overexpress SCARF1 in the AR group to observe whether overexpression of SCARF1 exerted a protective effect on AR after liver transplantation. Five days after liver transplantation (Figure 2A,B), the expression of SCARF1 in the AR group was significantly lower than that in the IT group. Meanwhile, the expression of SCARF1 in the AR + Ctrl group was not significantly altered compared to that in the AR group, whereas SCARF1 expression in the AR + OE group was significantly higher than that in the AR group. HE staining (Figure 2C) revealed that in comparison with the IT group, the liver tissue of the AR group exhibited typical AR markers, including a large number of inflammatory cell infiltration, hepatocyte necrosis, fibrous tissue hyperplasia and a significantly increased AR index (Figure 2D). Concurrently, compared to the AR group, the above indexes (Figure 2D) were not significantly altered in the AR + Ctrl group. In the AR + OE group, the degree of AR (Figure 2C) in liver tissue was significantly reduced, and the AR index (Figure 2D) was also significantly decreased. Liver function markers were detected in each group (Figure 2E,F). Compared to levels in the IT group, serum ALT and AST levels in the AR group were significantly elevated. There was no significant change in these values in the AR + Ctrl group compared to those of the AR group; however, ALT and AST levels in the AR + OE group were significantly lower than those in the AR group. We further observed the expression of SCARF1 (Figure 2G,H) and the histopathological changes (Figure 2I), AR score (Figure 2J), and liver function markers (Figure 2K,L) in each group 15 days after liver transplantation. The results exhibited the same trend 5 days after liver transplantation. In general, we observed that overexpression of SCARF1 in KCs alleviated the degree of AR after liver transplantation and improved the level of liver function markers, all of which were beneficial for alleviating the occurrence of AR after liver transplantation.

### 3.3 SCARF1 overexpression in KCs promoted a microenvironment supportive of IT after liver transplantation

In the process of AR of liver transplantation, the production of apoptotic T cells and the secretion of inflammatory factors are two very important factors. KCs act as the primary phagocytes and are an important source of inflammatory factors in the liver. Therefore, we further examined whether SCARF1 present in KCs could affect the timely clearance of apoptotic cells and the inhibition of inflammatory factor secretion after liver transplantation.

**FIGURE 3:**
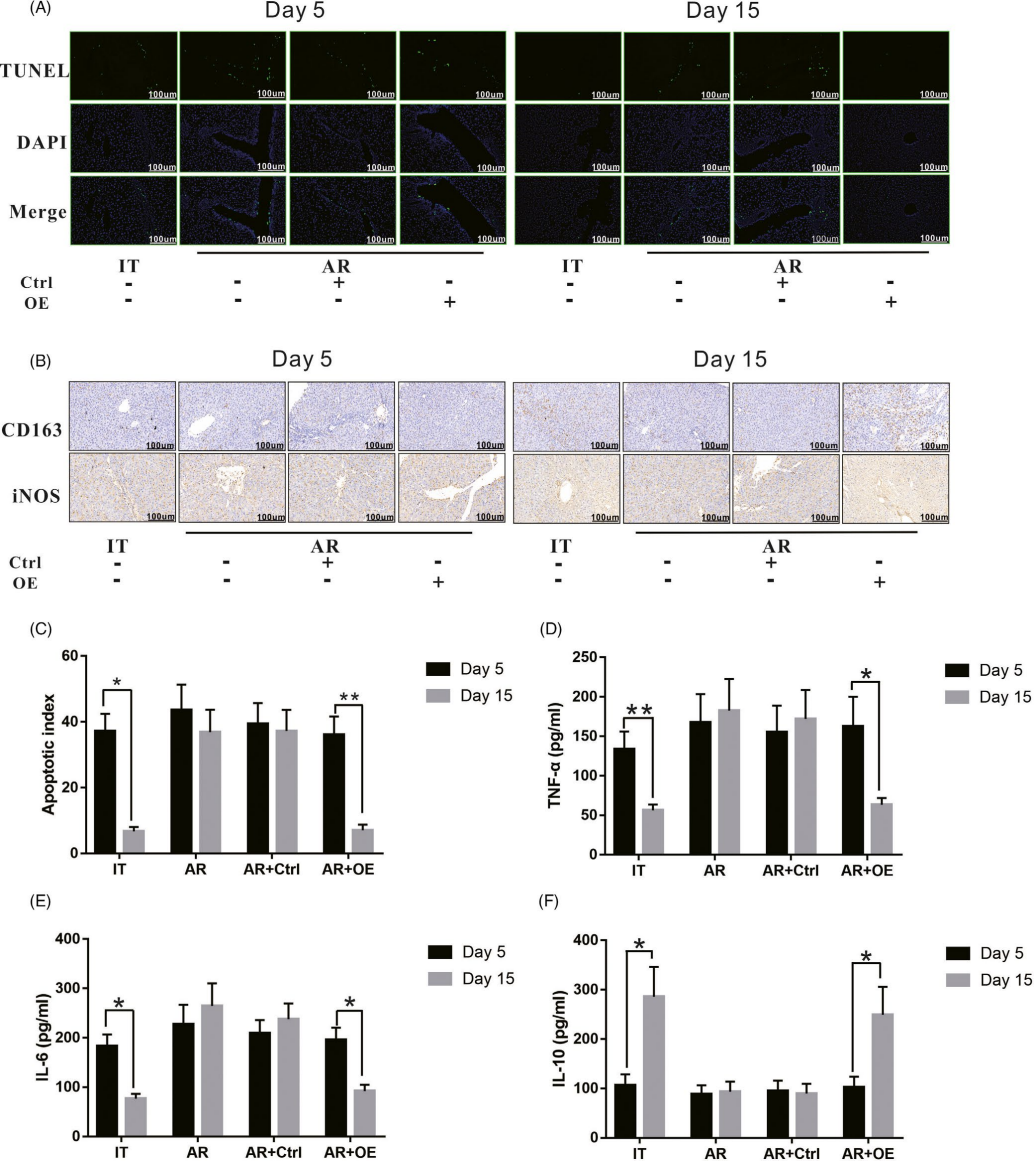
Effects of overexpression of SCARF1 in KCs on the clearance of apoptotic cells, the polarization of KCs, and the secretion of inflammatory factors in an acute rejection model of liver transplantation. A, The number of apoptotic cells in liver tissues from each group was detected via TUNEL staining 5 and 15 days after liver transplantation (200×). B, Polarization of KCs in liver tissue of rats of each group was detected via immunohistochemical staining (200×). C, Relative apoptotic index of each group. D, Level of serum TNF‐α in each group. E, Level of serum IL‐6 in each group. F, Level of serum IL‐6 in each group. *: *P* < .05, **: *P* < .01

Five days after liver transplantation, TUNEL staining revealed that a large number of apoptotic cells appeared in each group of liver tissues, particularly in proximity to blood vessels (Figure 3A,C). On the 15th day after liver transplantation, the number of apoptotic cells in the IT group was significantly decreased. There were still a large number of apoptotic cells in the liver tissues of the AR and the AR + Ctrl groups; however, the number of apoptotic cells in the liver tissue of the AR + OE group was significantly decreased (Figure 3A,C). These results suggest that a large number of apoptotic T cells appear in the liver tissue in both the tolerance model and the AR model in the early stage after liver transplantation. In the late stage after liver transplantation, the apoptotic cells present in the liver tissue of the tolerant model can be quickly removed due to normal apoptosis clearance. In the AR model, the low expression of SCARF1 in KCs resulted in an impairment of this clearance ability and the accumulation of apoptotic cells. When SCARF1 was overexpressed in KCs, the apoptotic clearance was improved, and the apoptotic cells were removed in a timely manner.

We next detected the polarization state of KCs and the secretion of inflammatory factors. As shown in Figure 3B, at 5 days after liver transplantation, immunohistochemical staining (Figure 3B) revealed that in all groups, iNOS‐positive (M1 type) KC infiltration was the major type, while CD163‐positive (M2 type) KC infiltration was rare. On the 15th day after liver transplantation, in the IT group, the infiltration of iNOS‐positive KCs was significantly decreased, and the infiltration of CD163 positive KCs was significantly increased. In the AR and AR + Ctrl groups, the infiltration of iNOS‐positive KCs was still predominant, and the infiltration of CD163‐positive KCs exhibited no obvious change. However, in the AR + OE group, the infiltration of CD163‐positive KCs was significantly increased, while the infiltration of iNOS‐positive KCs was significantly decreased. Concurrently, ELISA results indicated (Figure 3D‐F) that in all groups, the serum levels of TNF‐α and IL‐6 were higher and the levels of IL‐10 were lower at 5 days after liver transplantation. On the 15th day after liver transplantation, the level of IL‐10 in the IT group was significantly increased, while the levels of TNF‐α and IL‐6 were significantly decreased. In the AR and AR + Ctrl groups, TNF‐α and IL‐6 remained at high levels, and IL‐10 did not increase significantly. In the AR + OE group, IL‐10 levels were significantly increased, while TNF‐α and IL‐6 were significantly decreased.

Our results demonstrate that overexpression of SCARF1 in KCs can reduce the number of apoptotic cells in an AR model of liver transplantation, promote the transformation of KCs from M1 to M2 type, reduce the secretion of TNF‐α and IL‐6, and increase the secretion of IL‐10, all of which are conducive to the formation of an IT microenvironment after liver transplantation.

### 3.4 SCARF1‐mediated enhancement of phagocytosis in KCs is required for apoptotic cell clearance

**FIGURE 4:**
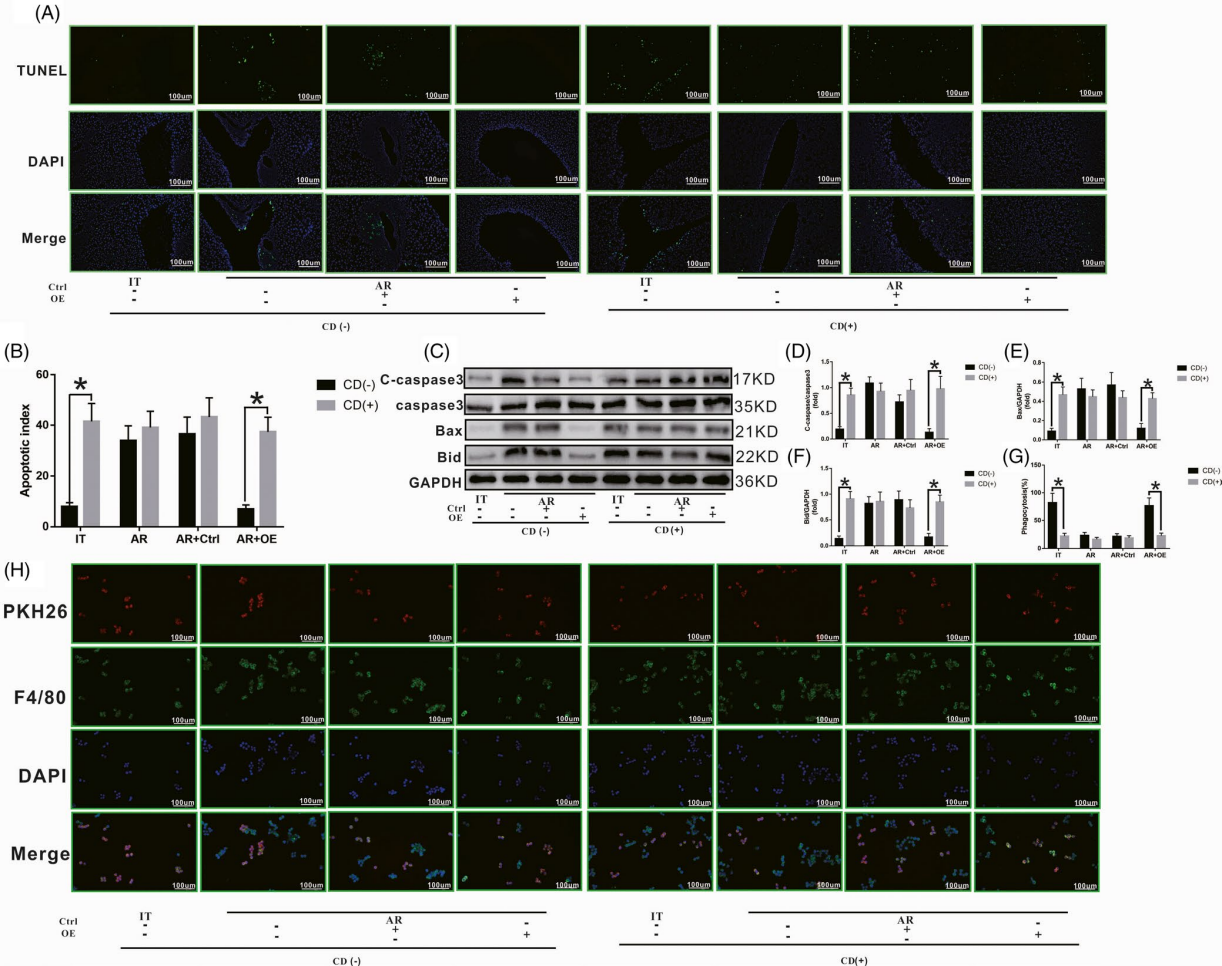
SCARF1‐mediated enhancement of phagocytosis of apoptotic cells by KCs. A, The number of apoptotic cells in liver tissues from rats of each group with or without Cytochalasin D treatment were detected via TUNEL staining. B, Relative number of apoptotic cells in liver tissues from each group. C, Levels of caspase3, Bax and Bid in the suspended apoptotic cells of each group. D, E and F, Relative expression of caspase3, Bax and Bid in each group. G, The number of apoptotic cells phagocytized by KCs with or without Cytochalasin D treatment was detected via immunofluorescence staining. H, Relative phagocytosis ratio in each group. *: *P* < .05

We next sought to confirm that the decrease in apoptotic cells in liver tissue after overexpression of SCARF1 is due to the enhanced phagocytic clearance of apoptotic cells by KCs and not because of a decrease in apoptotic cells themselves. Cytochalasin D (CD) was used to block the phagocytic function of KCs and to observe whether SCARF1‐mediated apoptosis clearance was altered. Fifteen days after liver transplantation (Figure 4A,B), after phagocytosis was blocked, the number of apoptotic cells in the IT group increased significantly compared to that prior to blocking of phagocytosis. Concurrently, after phagocytosis was blocked, the number of apoptotic cells in the liver tissue of the AR and AR + Ctrl groups did not change significantly compared to that prior to blocking. It should be noted that after phagocytosis was blocked, apoptotic cells in the liver tissue of the AR + OE group were also significantly increased.

KCs were then isolated and co‐cultured with apoptotic T cells in vitro. At 24 hours after co‐culture, the suspended apoptotic cells were collected. As shown in Figure 4C‐F, after phagocytosis was blocked, the levels of c‐caspase3, Bax and Bid in the IT group increased significantly compared to that prior to blocking. Meanwhile, after phagocytosis was blocked, the levels of c‐caspase3, Bax and Bid in the AR group and AR + Ctrl group did not change significantly compared to that observed prior to blocking. However, after phagocytosis was blocked, the levels of c‐caspase3, Bax and Bid in the AR + OE group were also significantly increased. At 24 hours after co‐culture, adherent KCs were also collected. As shown in Figure 4G,H, apoptotic cells were labelled with PKH26 (red) and KCs were labelled with F4/80 (green). After blocking phagocytosis, the number of apoptotic cells phagocytized by KCs (level of co‐localization of red fluorescence and green fluorescence) decreased significantly in the IT group. Meanwhile, after phagocytosis was blocked, the number of apoptotic cells phagocytized by KCs in the AR group and AR + Ctrl group did not change significantly compared to that observed prior to blocking. However, after phagocytosis was blocked, the number of apoptotic cells phagocytized by KCs in the AR + OE group also decreased significantly.

In summary, SCARF1‐mediated enhancement of phagocytosis in KCs is required for apoptotic cell clearance after liver transplantation.

### 3.5 SCARF1‐mediated enhancement of phagocytosis in KCs promotes M2 polarization

Previous studies have demonstrated that KCs phagocytize apoptotic cells, and this is an important means for inducing cell polarity remodelling. Our results also revealed that the polarization state of KCs was related to the different phagocytic functions of apoptotic cells. To further clarify whether SCARF1‐mediated enhancement of KCs phagocytosis can promote the polarization transition from M1 to M2, CD was used to block the phagocytic function of KCs to determine whether the polarization state of these KCs was altered.

**FIGURE 5:**
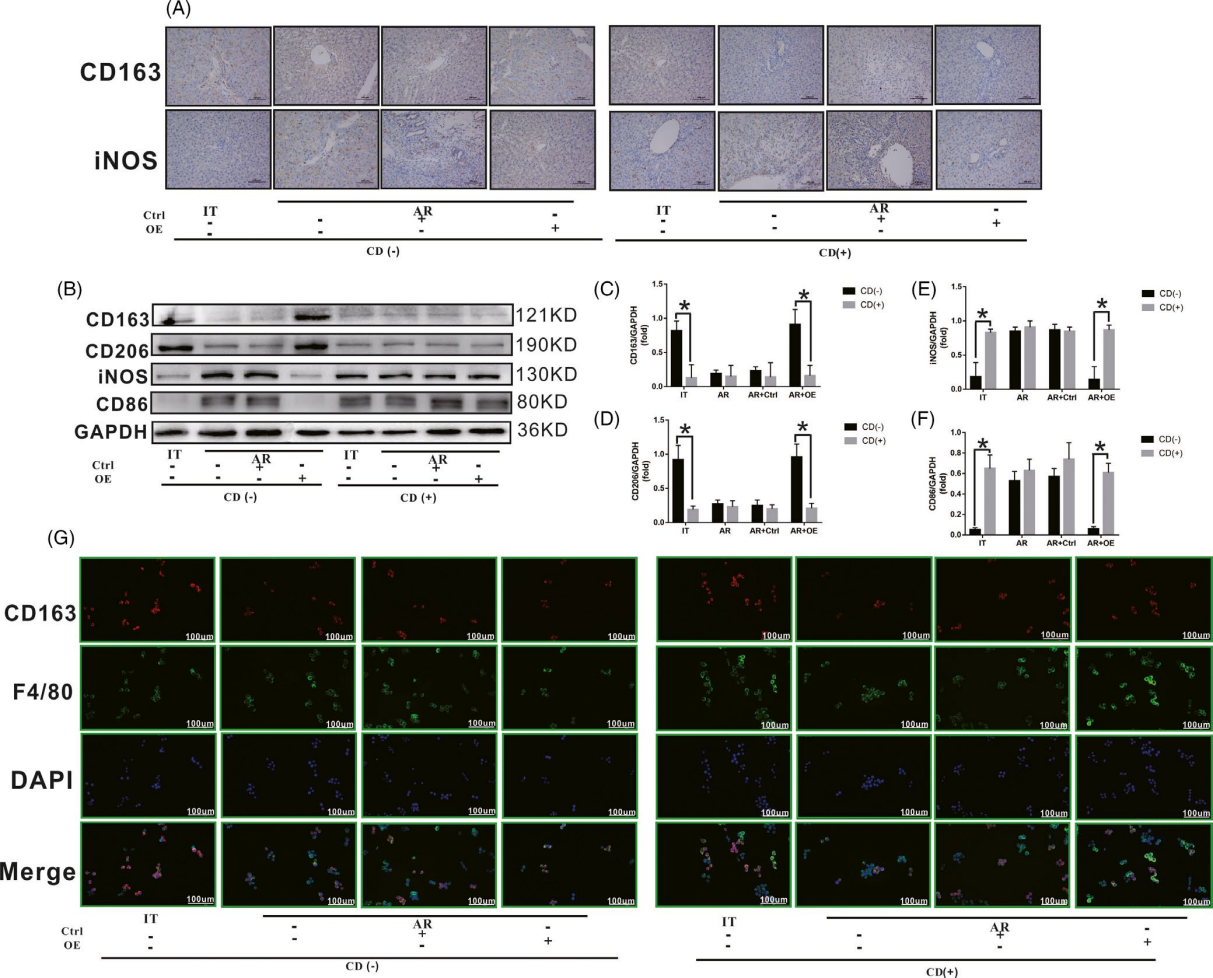
Effect of SCARF1‐mediated enhancement of phagocytosis on the regulation of KC polarization state. A, The numbers of CD163‐ or iNOS‐positive KCs in liver tissues from each group with or without Cytochalasin D treatment were detected via immunohistochemical staining. B, The levels of CD163, CD206, iNOS and CD86 in liver tissue of rats of each group with or without Cytochalasin D treatment were detected via WB. C, D, E and F, Relative expression of CD163, CD206, iNOS and CD86 in each group. G, The number of CD163 and F4/80 double‐positive KCs in each group with or without cytochalasin D treatment was detected via immunofluorescence staining. *: *P* < .05

At 15 days after liver transplantation (Figure 5A), after phagocytosis was blocked in the IT group, the number of CD163‐positive KCs decreased significantly, and the number of NOS‐positive KCs increased significantly compared to that in the untreated cells. Concurrently, after the phagocytosis was blocked in the AR and AR + Ctrl groups, both the number of CD163‐positive KCs and the number of NOS‐positive KCs did not change significantly compared to the untreated cells. However, after phagocytosis was blocked in the AR + OE group, the number of CD163‐positive KCs decreased significantly and the number of NOS‐positive KCs increased significantly compared to these numbers in the untreated cell populations. Meanwhile, M1‐related proteins (iNOS and CD86) and M2‐related proteins (CD206 and CD163) were detected in each group of liver KCs. As shown in Figure 5B‐F, after phagocytosis was blocked in the IT group, the levels of iNOS and CD86 increased significantly and the levels of CD206 and CD163 decreased significantly. After the phagocytosis was blocked in the AR and AR + Ctrl groups, the levels of iNOS, CD86, CD206 and CD163 did not change significantly compared to those in cells that were not treated with phagocytosis blockers. However, after phagocytosis was blocked in the AR + OE group, the levels of iNOS and CD86 increased significantly and the levels of CD206 and CD163 decreased significantly.

KCs were then isolated and co‐cultured with apoptotic T cells in vitro. As shown in Figure 5G, at 24 hours after co‐culture, KCs were labelled with CD163 (red) and F4/80 (green). After blocking of phagocytosis, the number of M2 KCs (level of co‐localization of red fluorescence and green fluorescence) decreased significantly in the IT group. Meanwhile, after the phagocytosis was blocked, the number of M2 KCs in the AR group and AR + Ctrl group did not change significantly compared to the unblocked cells. However, after phagocytosis was blocked, the number of M2 KCs in the AR + OE group also decreased significantly.

These results confirmed our hypothesis that SCARF1‐mediated enhancement of KCs phagocytosis was beneficial for the transformation of KCs from the M1 to the M2 phenotype.

### 3.6 The calcium‐dependent PI3K‐AKT‐STAT3 signalling pathway exhibited increased activity during phagocytosis

**FIGURE 6:**
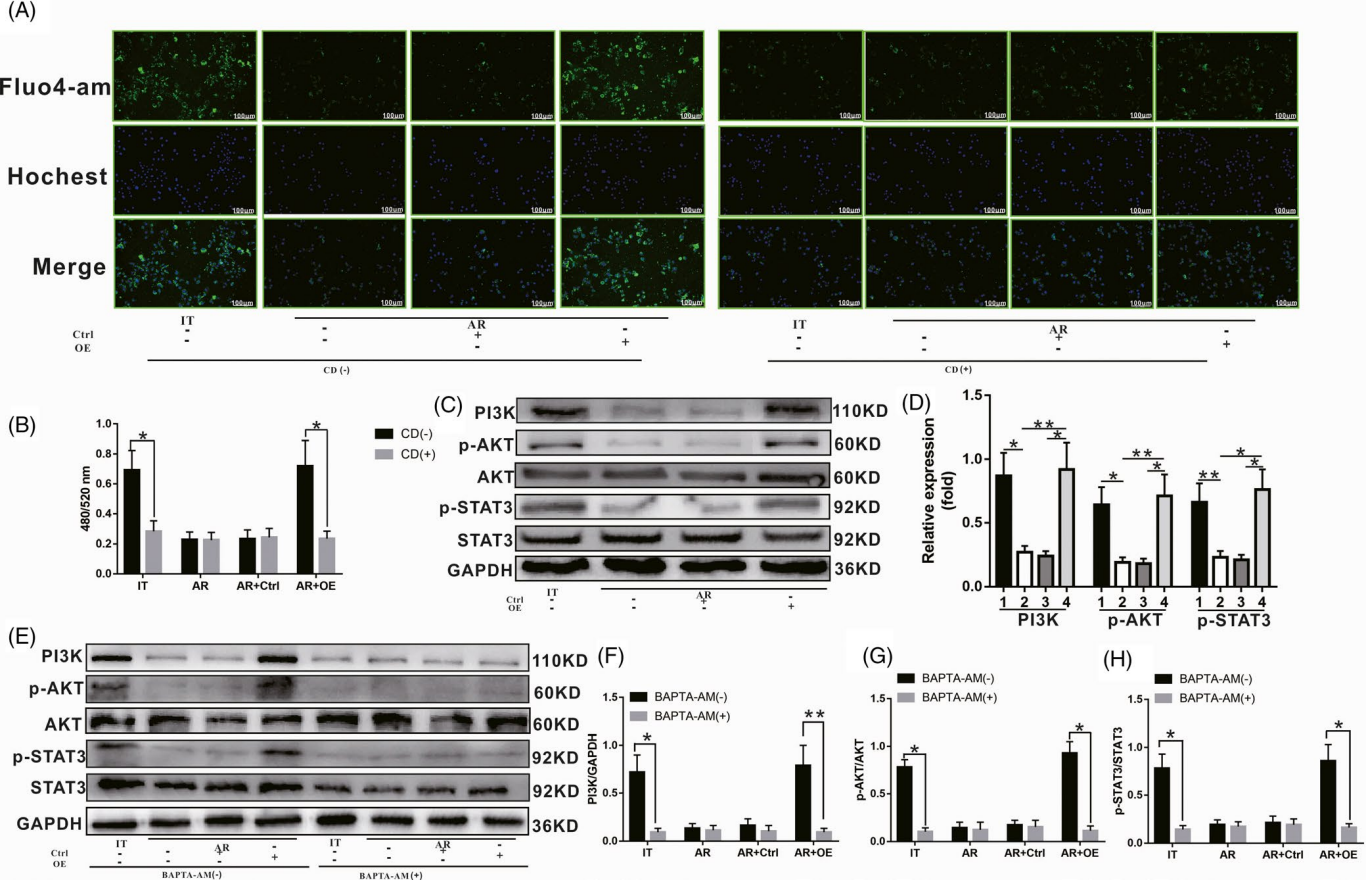
Effect of SCARF1‐mediated enhancement of phagocytosis on the calcium‐dependent PI3K‐AKT‐STAT3 signalling pathway. A, The concentration of calcium was detected via immunofluorescence, where intracellular calcium was labelled by Fluo‐4AM (green) and the nucleus was labelled by Hoechst. B, The relative concentration of calcium in each group was detected using a fluorescence microplate reader. C, The levels of PI3K, p‐AKT and p‐STAT3 in each group were detected via WB. D, Relative expression of PI3K, p‐AKT and p‐STAT3 in each group. E, The levels of PI3K, p‐AKT and p‐STAT3 in each group with or without BAPTA‐AM treatment. F, G and H, Relative expression of PI3K, p‐AKT and p‐STAT3 in each group with or without BAPTA‐AM treatment. *: *P* < .05, **: *P* < .01

The change in intracellular calcium concentration is an important event in the process of phagocytosis. Previous studies also determined that an increase in intracellular calcium concentration can promote the activation of the PI3K‐AKT signalling pathway. We speculate that the calcium‐dependent PI3K‐AKT signalling pathway may play an important role in SCARF1‐mediated transformation of KCs induced by enhanced phagocytosis. First, at 24 hours after co‐culture, the concentration of calcium in KCs was detected via immunocytochemistry and using a fluorescence microplate reader. As shown in Figure 6A,B, compared to the IT group, the calcium concentration in KCs in the AR and AR + Ctrl groups was decreased significantly. However, compared to the AR and AR + Ctrl groups, the calcium concentration in KCs in the AR + OE group was increased significantly. After blocking of phagocytosis, the calcium concentration in KCs in the IT group decreased significantly compared to that in unblocked cells. Meanwhile, the calcium concentration in KCs in the AR and AR + Ctrl groups did not change significantly compared to that in untreated cells. However, the calcium concentration in KCs in the AR + OE group was also significantly decreased compared to that in untreated cells.

We further examined the effect of calcium concentration on the activation of the PI3K‐AKT‐STAT3 signalling pathway. As shown in Figure 6C,D, the levels of PI3K, p‐AKT and p‐STAT3 in each group were consistent with the change in calcium concentration. To confirm that the activation of the PI3K‐AKT‐STAT3 signalling pathway is directly regulated by intracellular calcium, the calcium antagonist BAPTA‐AM was used to block intracellular calcium. As shown in Figure 6E‐H, after blocking intracellular calcium, the levels of PI3K, p‐AKT and p‐STAT3 in KCs in the IT group decreased significantly compared to those of the untreated cells. Meanwhile, the levels of PI3K, p‐AKT and p‐STAT3 in KCs in the AR and AR + Ctrl groups did not change significantly compared to those of the untreated cells. However, the levels of PI3K, p‐AKT and p‐STAT3 in KCs in the AR + OE group also decreased significantly compared to those of the untreated cells.

In conclusion, our experimental results demonstrate that SCARF1‐mediated enhancement of phagocytosis promotes an increase in calcium concentration in KCs, and this increase in calcium concentration further activates the PI3K‐AKT‐STAT3 signalling pathway, thus identifying a potential mechanism underlying SCARF1‐mediated enhancement of phagocytosis to promote the polarization state change of KCs.

### 3.7 SCARF1‐mediated calcium‐dependent PI3K‐AKT‐STAT3 signalling pathway promotes M2 polarization of KCs

To further confirm the effect of the calcium‐dependent PI3K‐AKT‐STAT3 signalling pathway on the polarization of KCs, a PI3K inhibitor (IPI‐549), an AKT activity inhibitor (GSK2141795) and a STAT3 phosphorylation inhibitor (Stattic) were all used to inhibit PI3K and the phosphorylation of AKT and STAT3, respectively. Thereafter, we observed the changes in the entire signalling pathway and the polarization state of KCs in the presence and absence of the above inhibitors.

**FIGURE 7:**
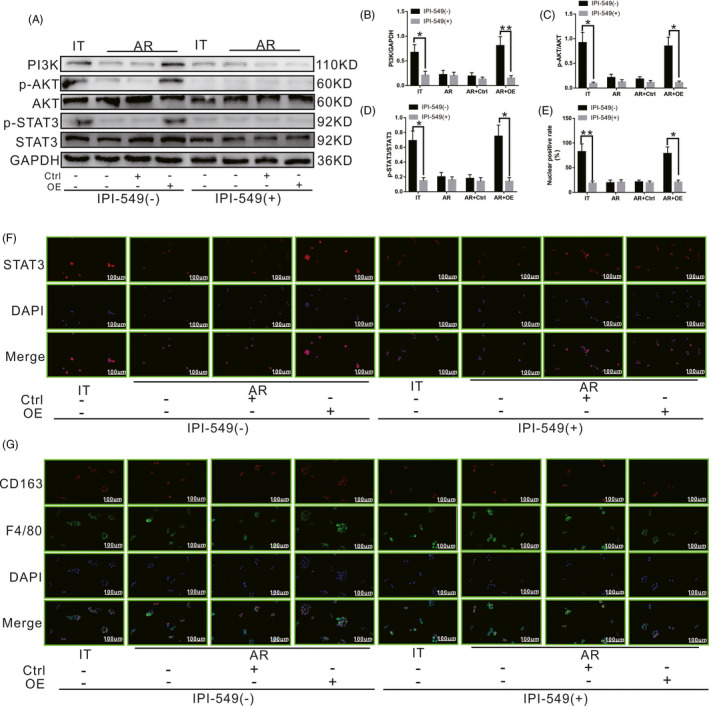
Effect of the SCARF1‐mediated calcium‐dependent PI3K‐AKT‐STAT3 signalling pathway on the polarization state of KCs in the presence or absence of the PI3K inhibitor IPI‐549. A, The levels of PI3K, p‐AKT and p‐STAT3 in each group with or without IPI‐549 treatment were detected via WB. B, C and D, Relative expression of PI3K, p‐AKT and p‐STAT3 in each group with or without IPI‐549 treatment. E, Relative nuclear expression rate of STAT3 in each group with or without IPI‐549 treatment. F, STAT3 positioning in each group with or without IPI‐549 treatment was detected using immunocytochemistry. G, The numbers of CD163 and F4/80 double‐positive KCs in each group with or without IPI‐549 treatment were detected via immunofluorescence staining. *: *P* < .05, **: *P* < .01

As shown in Figure 7A‐D, after inhibiting PI3K using IPI‐549, the levels of PI3K, p‐AKT and p‐STAT3 in KCs in the IT group were decreased significantly compared to those of untreated cells. Meanwhile, the levels of PI3K, p‐AKT and p‐STAT3 in KCs in the AR and AR + Ctrl groups did not change significantly compared to those of untreated cells. However, the levels of PI3K, p‐AKT and p‐STAT3 in KCs in the AR + OE group were also significantly decreased compared to those of untreated cells. Activated STAT3 enters the nucleus after phosphorylation, and based on this, the nuclear penetration of STAT3 was also detected using immunocytochemistry. As shown in Figure 7E,F, after inhibiting PI3K using IPI‐549, the nuclear‐positive rate for STAT3 in KCs in the IT group decreased significantly compared to that of untreated cells. Meanwhile, the nuclear‐positive rate for STAT3 in KCs in the AR group and AR + Ctrl group did not change significantly compared to that of untreated cells. However, the nuclear‐positive rate for STAT3 in KCs in the AR + OE group was also significantly decreased compared to that of untreated cells. As shown in Figure 7G, at 24 hours after co‐culture, KCs were labelled with CD163 (red) and F4/80 (green). After inhibiting PI3K using IPI‐549, the number of M2 KCs (level of co‐localization of red fluorescence and green fluorescence) decreased significantly in the IT group compared to that of untreated cells. Meanwhile, the number of M2 KCs in the AR group and AR + Ctrl group did not change significantly compared to that of untreated cells. However, the number of M2 KCs in the AR + OE group was also significantly decreased compared to that of untreated cells. Our results demonstrate that PI3K is located upstream of the entire signalling pathway, and inhibition of PI3K can block the activation of AKT and STAT3 and the M2 polarization of KCs.

**FIGURE 8:**
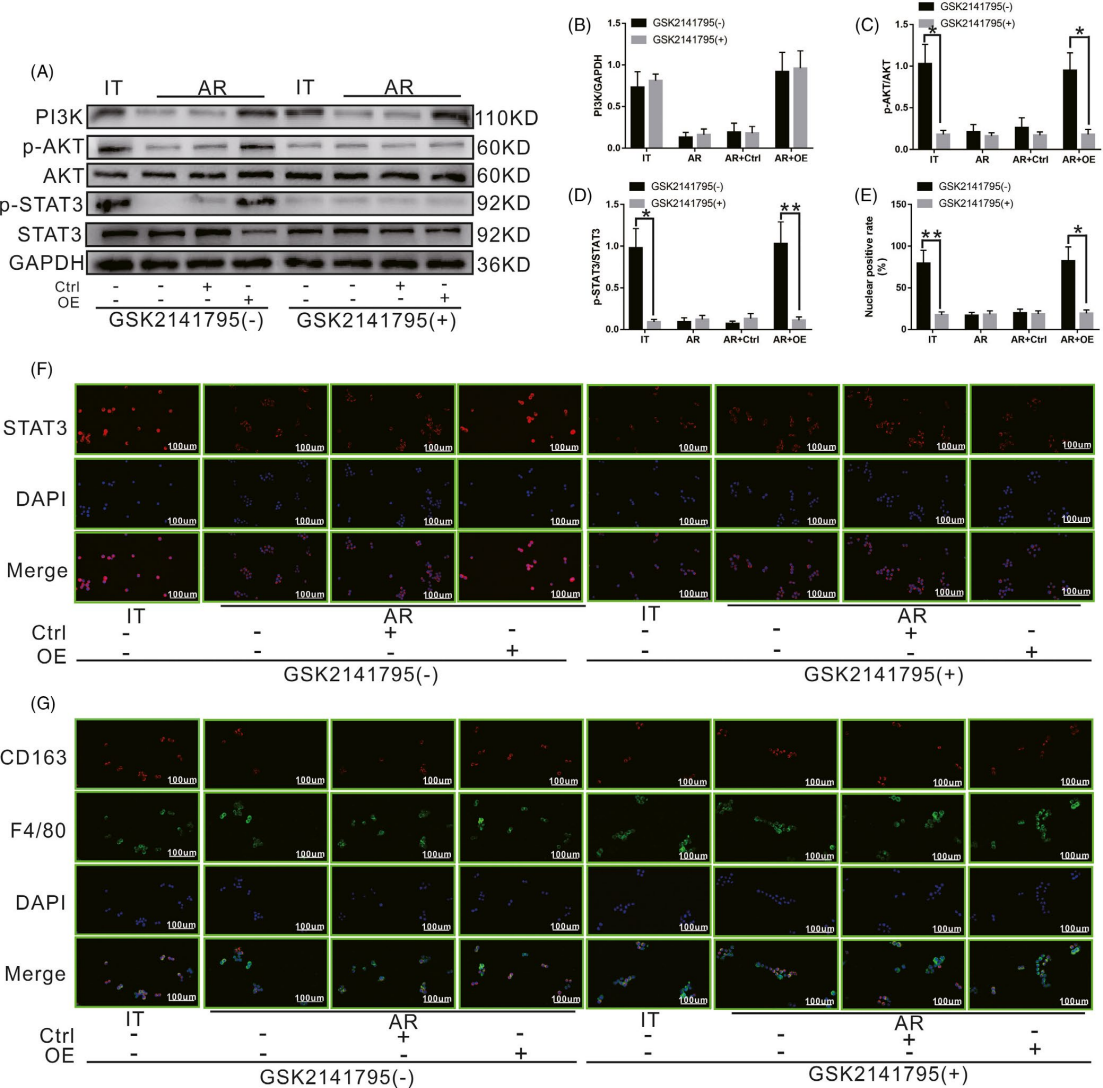
Effect of the SCARF1‐mediated calcium‐dependent PI3K‐AKT‐STAT3 signalling pathway on the polarization state of KCs in the presence or absence of the AKT phosphorylation inhibitor GSK2141795. A, The levels of PI3K, p‐AKT and p‐STAT3 in each group with or without GSK2141795 treatment were detected via WB. B, C and D, Relative expression of PI3K, p‐AKT and p‐STAT3 in each group with or without GSK2141795 treatment. E, Relative nuclear expression rate of STAT3 in each group with or without GSK2141795 treatment. F, STAT3 positioning in each group with or without GSK2141795 treatment was detected via immunocytochemistry. G, The numbers of CD163 and F4/80 double‐positive KCs in each group with or without GSK2141795 treatment were detected via immunofluorescence staining. *: *P* < .05, **: *P* < .01

As shown in Figure 8A‐D, after inhibiting the activity of AKT using GSK2141795, with the exception of the level of PI3K, the levels of p‐AKT and p‐STAT3 in KCs in the IT group decreased significantly compared to those of untreated cells. Meanwhile, the levels of PI3K, p‐AKT and p‐STAT3 in KCs in the AR and AR + Ctrl groups did not change significantly compared to those of untreated cells. However, except for the level of PI3K, the levels of p‐AKT and p‐STAT3 in KCs in the AR + OE group also decreased significantly compared to those of untreated cells. Nuclear penetration by STAT3 was detected via immunocytochemistry. As shown in Figure 8E,F, after inhibiting the activity of AKT using GSK2141795, the nuclear‐positive rate of STAT3 in KCs in the IT group decreased significantly compared to that of untreated cells. Meanwhile, the nuclear‐positive rate of STAT3 in KCs in the AR group and AR + Ctrl group did not change significantly compared to that of untreated cells. However, the nuclear‐positive rate of STAT3 in KCs in the AR + OE group also decreased significantly compared to that of untreated cells. As shown in Figure 8G, at 24 hours after co‐culture, KCs were labelled with CD163 (red) and F4/80 (green). After inhibiting the activity of AKT using GSK2141795, the number of M2 KCs decreased significantly in the IT group compared to that of untreated cells. Meanwhile, the number of M2 KCs in the AR group and AR + Ctrl group did not change significantly compared to that of untreated cells. However, the number of M2 KCs in the AR + OE group also decreased significantly compared to that of untreated cells. Our results revealed that inhibition of AKT phosphorylation could block STAT3 phosphorylation and the M2 polarization of KCs, even if PI3K activation was not affected.

**FIGURE 9:**
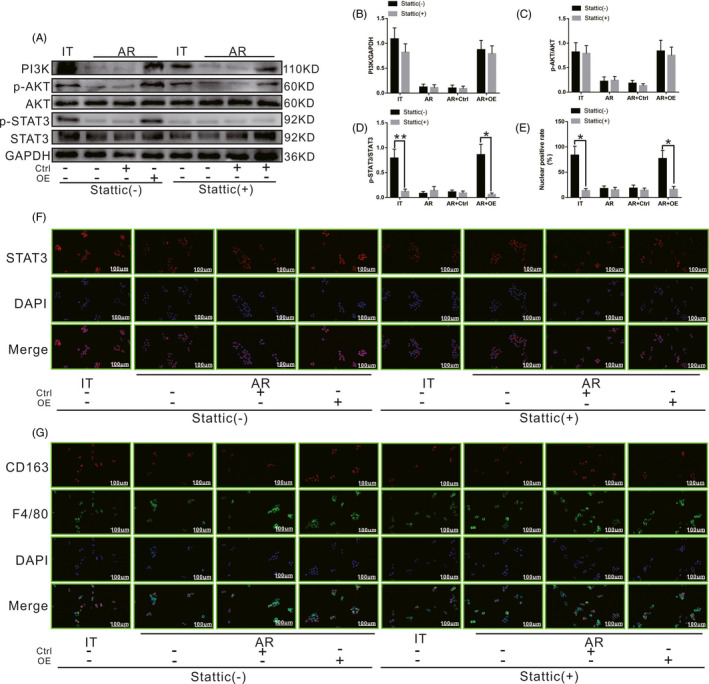
Effect of the SCARF1‐mediated calcium‐dependent PI3K‐AKT‐STAT3 signalling pathway on the polarization state of KCs in the presence or absence of the STAT3 phosphorylation inhibitor Stattic. A, The levels of PI3K, p‐AKT and p‐STAT3 in each group with or without Stattic treatment were detected via WB. B, C and D, Relative expression of PI3K, p‐AKT and p‐STAT3 in each group with or without Stattic treatment. E, Relative nuclear expression rate of STAT3 in each group with or without Stattic treatment. F, STAT3 positioning in each group with or without Stattic treatment was detected via immunocytochemistry. G, The numbers of CD163 and F4/80 double‐positive KCs in each group with or without Stattic treatment were detected via immunofluorescence staining. *: *P* < .05, **: *P* < .01

As shown in Figure 9A‐D, after inhibiting phosphorylation of STAT3 using Stattic, the level of p‐STAT3 in KCs in the IT group decreased significantly compared to that of untreated cells, while the levels of PI3K and p‐AKT did not change significantly. Meanwhile, the levels of PI3K, p‐AKT and p‐STAT3 in KCs in the AR and AR + Ctrl groups did not change significantly compared to those of untreated cells. However, the level of p‐STAT3 in KCs in the AR + OE group also decreased significantly compared to that of untreated cells, while the levels of PI3K and p‐AKT did not change significantly. Nuclear penetration by STAT3 was detected via immunocytochemistry. As shown in Figure 9E,F, after inhibiting phosphorylation of STAT3 by Stattic, the nuclear‐positive rate for STAT3 in KCs in the IT group decreased significantly compared to that of untreated cells. Meanwhile, the nuclear‐positive rate of STAT3 in KCs in the AR group and AR + Ctrl group did not change significantly compared to that of untreated cells. However, the nuclear‐positive rate of STAT3 in KCs in the AR + OE group also decreased significantly compared to that of untreated cells. As shown in Figure 9G, after inhibiting phosphorylation of STAT3 using Stattic, the number of M2 KCs decreased significantly in the IT group compared to that of untreated cells. Meanwhile, the number of M2 KCs in the AR group and AR + Ctrl group did not change significantly compared to that of untreated cells. However, the number of M2 KCs in the AR + OE group also decreased significantly compared to that of untreated cells. Our results demonstrated that inhibiting phosphorylation of STAT3 can block the M2 polarization of KCs, even when the activation of PI3K and AKT is not affected.

In conclusion, our results confirm that SCARF1 promotes M2 polarization of KCs by promoting phagocytosis through the calcium‐dependent PI3K‐AKT‐STAT3 signalling pathway.

## 4 DISCUSSION

Recognition and elimination of apoptotic cells represent one of the important functions of macrophages. This progress is beneficial for maintaining immunity homeostasis and for forming IT against the autoantibodies.^19^, ^20^ If the process of elimination of apoptotic cells is defective, these apoptotic cells can accumulate and induce a ‘second necrosis’. In response to ‘second necrosis’, a mass of pro‐inflammatory factors including TNF‐α, IL‐1β and IFN‐γ can be released that subsequently induce inflammation or lead to the development of autoimmune disease.^21^, ^22^ According to a preliminary study, this process is applicable to multiple diseases, including AR induced by transplantation,^23^ oncogenesis ^24^ and autoimmune disease.^25^ Therefore, the key to inducing IT in the context of liver transplantation is to promote the timely clearance of apoptotic cells by phagocytes (primarily macrophages within the liver).

To activate the phagocytosis activity of macrophages, a series of receptors expressed on the surface of apoptotic cells were regarded to act as the key molecules. These molecules, including Tim3, Tim4 and BAI1, are termed ‘eat me’ signal molecules and play an important role in the elimination of apoptotic cells. Sada‐Ovalle I^26^ suggested that TIM‐3 can function as a molecule that participates in cell activation and the elimination of different pathogens. Tim4 was speculated to mediate the engulfment of apoptotic cells by peritoneal macrophages.^27^ Remarkably, due to a defect in SCARF1, although the expression of other ‘eat me’ signal molecules was normal, the clearance of apoptotic cells mediated by DC cells declined sharply.^3^ Our results demonstrate that the lack of SCARF1 in KCs is related to the occurrence of AR after liver transplantation. Overexpression of SCARF1 in KCs can effectively reduce the degree of AR after liver transplantation and can improve the level of liver function. Moreover, after overexpression of SCARF1 in KCs, the clearance of apoptotic cells due to enhanced phagocytosis was significantly increased, despite the observation that apoptosis was not significantly reduced. Therefore, it is likely that SCARF1 may play a more important role in the clearance of apoptotic cells, a process that may facilitate the formation of a microenvironment supportive of IT.

The M2 polarization of KCs is conducive to the formation of IT after liver transplantation, and it is also regarded as an important indicator of the formation of an IT microenvironment after liver transplantation.^28^ Our experiment demonstrated that after overexpression of SCARF1 in KCs, the polarization state of KCs transformed into the M2 type, and this was consistent with the decrease in apoptotic cells. Interestingly, previous studies have observed that the polarization state of KCs can transform into the M2 type in response to stimulation by apoptotic cells.^29^ After phagocytizing apoptotic cells, the change in the polarization state of KCs is a complex process that may involve activation of the TGF‐β/Smad signalling pathway,^30^ LC3‐associated phagocytosis,^31^ and activation of the aryl hydrocarbon receptor.^32^ As SCARF1 can promote the phagocytosis of apoptotic cells by KCs and can increase the number of M2 KCs in the liver after liver transplantation, we further evaluated whether the M2 polarization of KCs mediated by SCARF1 is facilitated by an enhancement in phagocytosis. We observed that after blocking the phagocytosis of KCs, the M2 transformation of KCs induced by SCARF1 was reversed. Therefore, it is likely that SCARF1‐mediated enhancement of KC phagocytosis is beneficial for the transformation of KCs from the M1 to the M2 phenotype.

STAT3 is thought to be involved in the regulation of M2 macrophage polarization. During the formation of an IT microenvironment, STAT3 is activated in a variety of ways, including through exosome stimulation,^33^ IL‐8 stimulation,^34^ IL‐10 stimulation^35^ and the MAPK signalling pathway.^36^, ^37^ Next, STAT3 promotes macrophage M2 polarization in the form of phosphorylation. AKT, a member of MAPKs, is also activated during macrophage phagocytosis. These results suggest that the AKT‐STAT3 pathway may provide an important connection between enhancing phagocytosis and promoting M2 polarization of macrophages.

Notably, the intracellular calcium concentration was significantly increased during the phagocytosis of APCs to clear apoptotic cells.^17^, ^38^, ^39^ Previous studies have shown that increased cytosolic calcium levels can promote the activation of the PI3K‐AKT signalling pathway.^40^, ^41^ Consistent with previous studies, our results revealed that after co‐culture with apoptotic cells for 24 hours, the intracellular calcium concentration and the PI3K‐AKT‐STAT3 signalling pathway were both increased. However, when intracellular calcium release was blocked, the intracellular calcium concentration of KCs did not increase, and the activity of the PI3K‐AKT‐STAT3 signalling pathway was also decreased, even under the stimulation of apoptotic cells. We further confirmed the effect of the calcium‐dependent PI3K‐AKT‐STAT3 signalling pathway on the polarization of KCs. Treatment with a PI3K inhibitor (IPI‐549), an AKT activity inhibitor (GSK2141795) or a STAT3 phosphorylation inhibitor (Stattic) resulted in the abolishment of SCARF1‐mediated phagocytic‐dependent M2 polarization of KCs. Therefore, we demonstrated that the calcium‐dependent PI3K‐AKT‐STAT3 signalling pathway promotes the polarization of KCs, and this process is initiated by SCARF1‐mediated enhancement of phagocytosis.

## 5 CONCLUSIONS

We believe that the upregulation of SCARF1 in KCs can suppress the damage caused by acute liver rejection and can promote IT.

## CONFLICT OF INTEREST

None.

## AUTHOR CONTRIBUTIONS

Jian‐ping Gong and Jin‐zheng Li designed the study and supervised the project. Xue‐Song Xu and Zhi‐hao Feng performed majority of the experimental work. Ding Cao and Hao Wu analysed the data. Xue‐Song Xu wrote the manuscript. Zhi‐hao Feng and Meng‐hao Wang proofread the manuscript. Jian‐ping Gong approved the final version of the manuscript. All authors have read and approved the final manuscript.

## Supporting information

Figure S1

Figure S2

Figure S3

Figure S4

Supplementary Material

## Data Availability

All data included in this study are available upon request by contacting the corresponding author.
