# Supplementary figures and images for: SCARF1 promotes M2 polarization of Kupffer cells via calcium‐dependent PI3K‐AKT‐STAT3 signalling to improve liver transplantation
Source: Cell Prolif. 2021 Mar 9;54(4):e13022. doi: 10.1111/cpr.13022 (PMC8016636; doi:10.1111/cpr.13022)

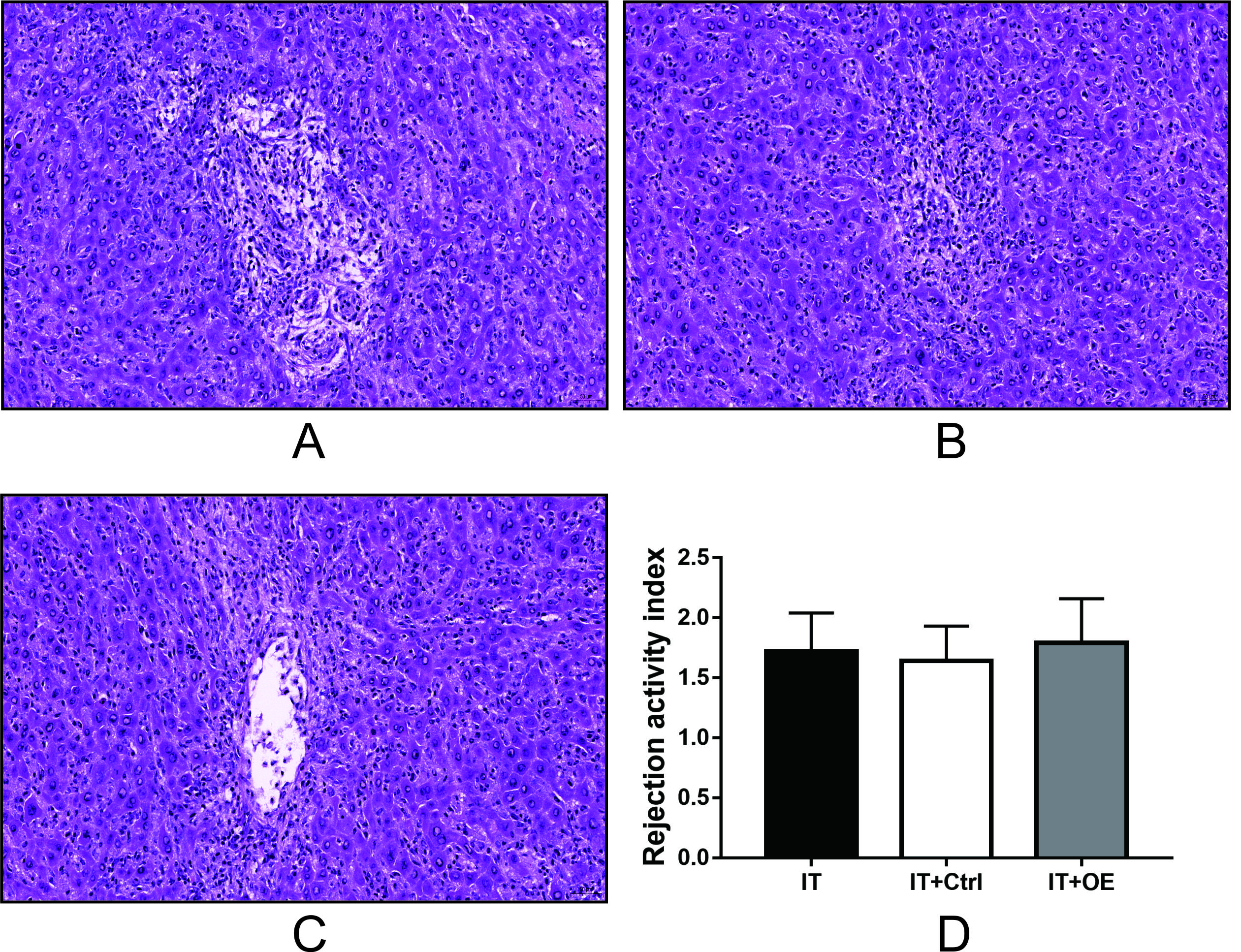

Supplement: Supplementary file 1 — Figure S1 [file CPR-54-e13022-s004.jpg]

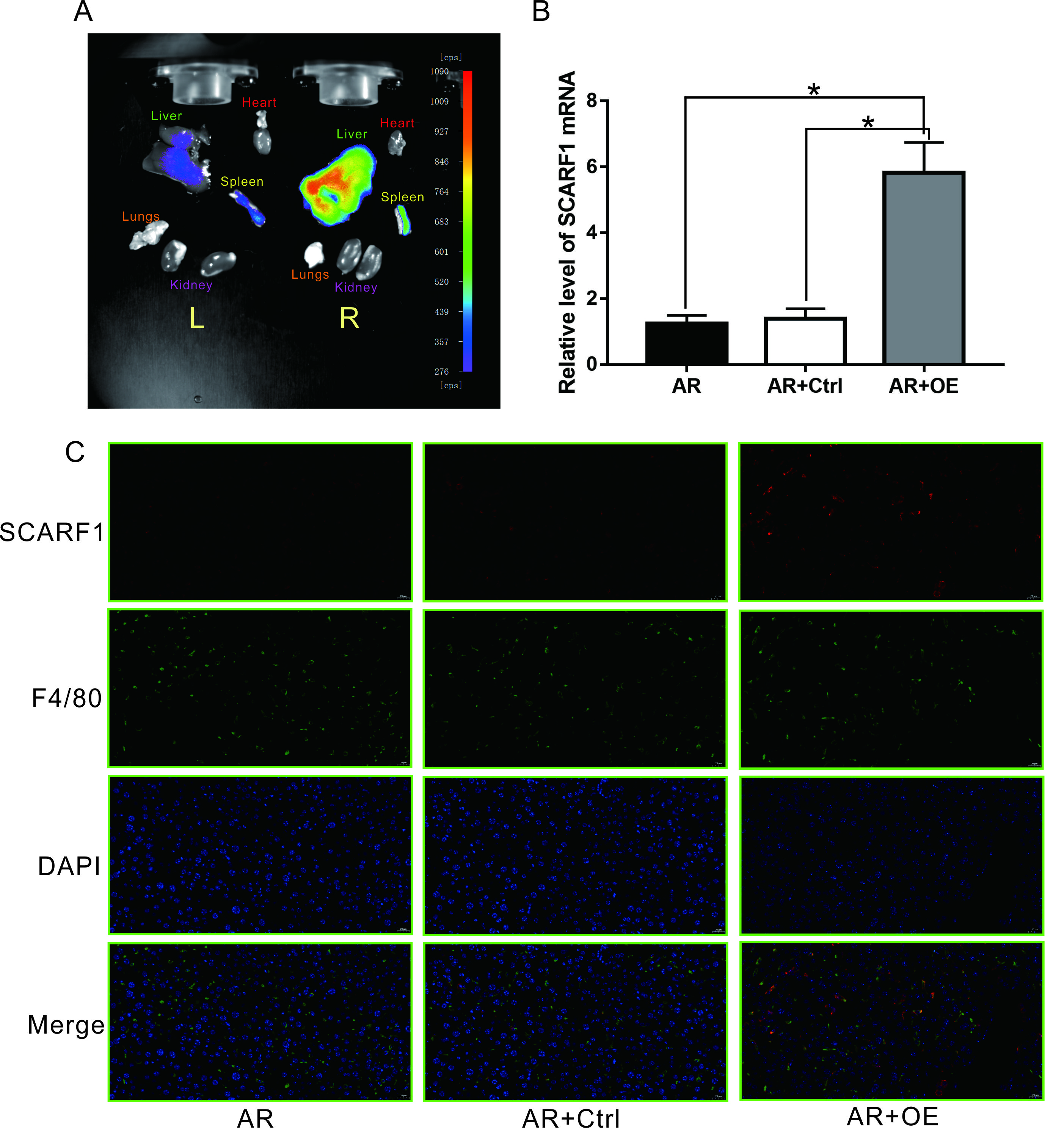

Supplement: Supplementary file 2 — Figure S2 [file CPR-54-e13022-s003.jpg]

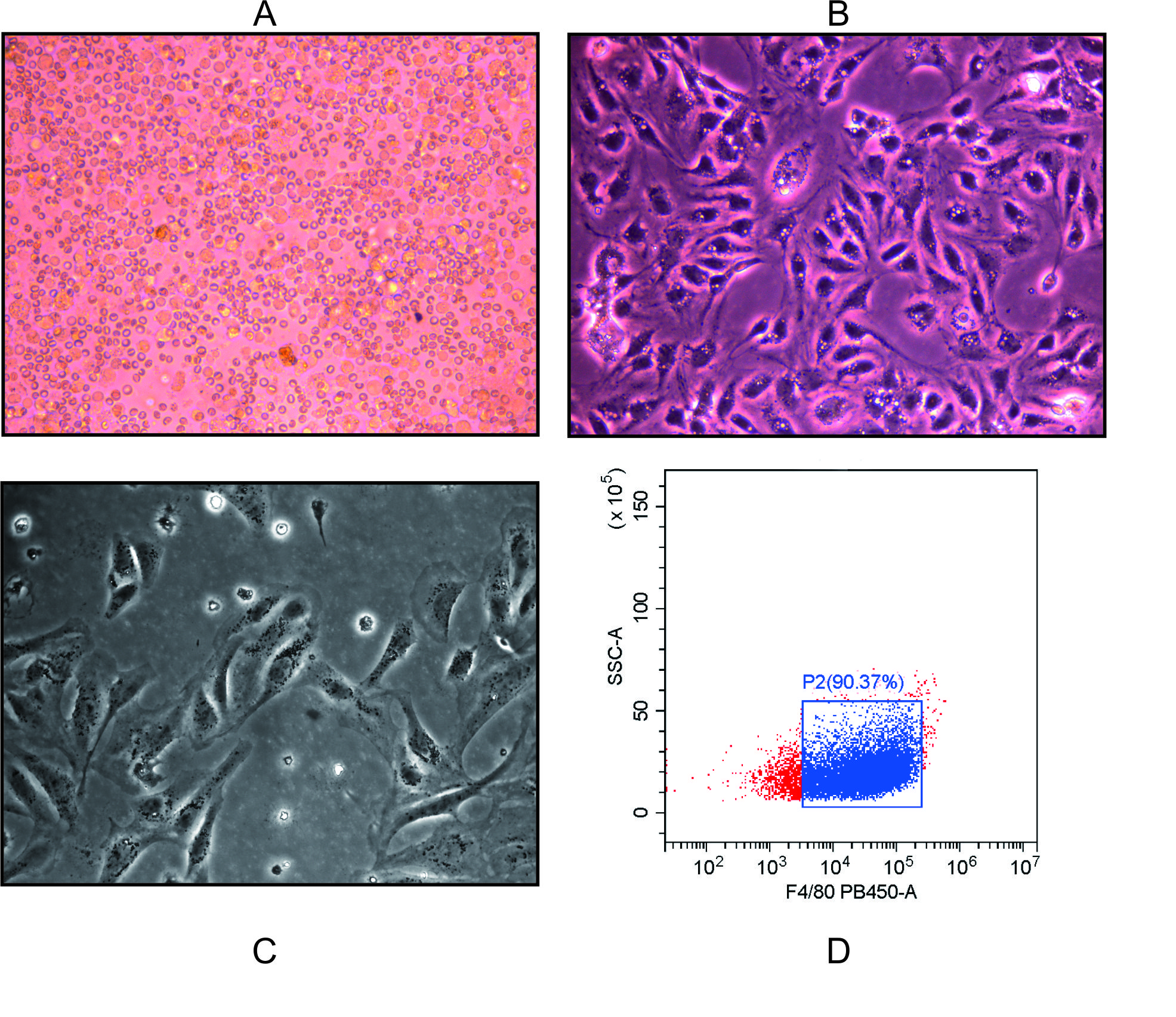

Supplement: Supplementary file 3 — Figure S3 [file CPR-54-e13022-s001.jpg]

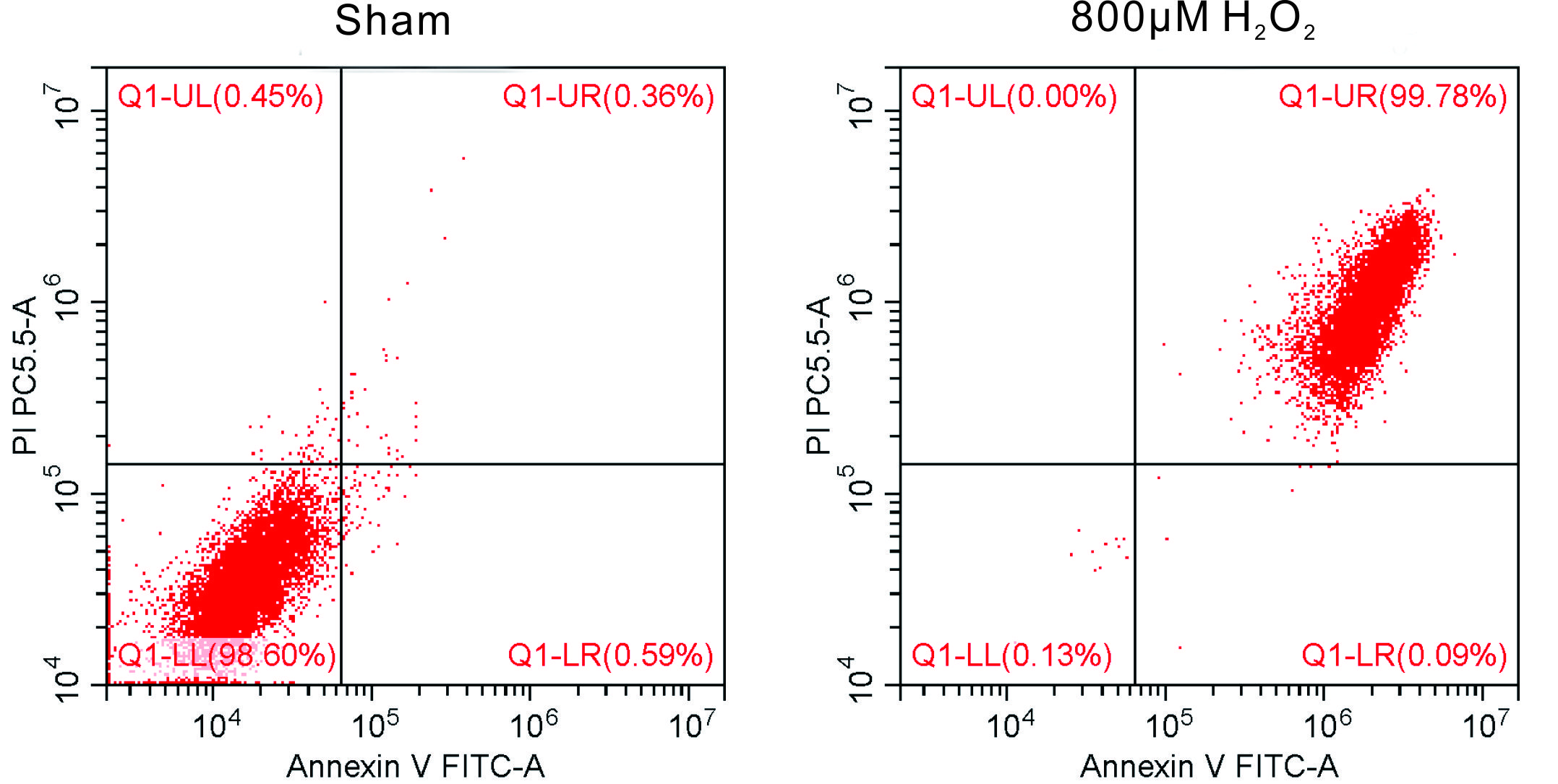

Supplement: Supplementary file 4 — Figure S4 [file CPR-54-e13022-s005.jpg]
