# Supplementary material for: SCARF1 promotes M2 polarization of Kupffer cells via calcium‐dependent PI3K‐AKT‐STAT3 signalling to improve liver transplantation
Source: Cell Prolif. 2021 Mar 9;54(4):e13022. doi: 10.1111/cpr.13022 (PMC8016636; doi:10.1111/cpr.13022)
Supplement: Supplementary file 5 — Supplementary Material [file CPR-54-e13022-s002.docx]

**Supplementary data**

**Figure S1. Pathological changes in liver tissue of rats in the IT group in the presence or absence of AAV transfection.**

**A:** Pathological changes in liver tissue of rats in the IT group (200×). **B:** Pathological changes in liver tissue of rats in the IT group with Ctrl- AAV (200×). **C:** Pathological changes in liver tissue of rats in the IT group with OE-AAV (200×). D: Acute rejection index for each group.

**Figure S2. Transfection efficiency of KCs *in vivo*. A:** Cell-specific identification of AAV transfection was achieved using living fluorescence imaging. L: cells treated with GdCl_3_. R: cells treated without GdCl_3_. **B:** effect of overexpression in each group was detected via RT-PCR. **C:** effect of overexpression in each group was detected via immunofluorescence (200×). *: *P* <0.05.

**Figure S3. Identification of the morphology, phagocytosis activity, and markers of KCs.** A. Cell morphology of KCs 2 h after culture (400×). B. Cell morphology of KCs 24 h after culture (400×). C. Results of the swallowing ink experiment (400×). D. The level of F4/80 in KCs was detected via flow cytometry.

**Figure S4. Induction effect of apoptotic cells.** The level of apoptosis with or without 100 µM H_2_O_2_ was detected via flow cytometry.
